# Treatment of a large through and through periapical lesion using guided tissue regeneration: A case report of 2 years follow‐up

**DOI:** 10.1002/ccr3.6405

**Published:** 2022-10-06

**Authors:** Buraikan Alajmi, Mohmed Isaqali Karobari, Omir Aldowah

**Affiliations:** ^1^ Endodontic Department at Adan Dental Center Ministry of Health Kuwait Kuwait; ^2^ Conservative Dentistry Unit, School of Dental Sciences Universiti Sains Malaysia, Health Campus Kubang Kerian Kota bharu Malaysia; ^3^ Department of Conservative Dentistry & Endodontics, Saveetha Dental College & Hospitals Saveetha Institute of Medical and Technical Sciences University Chennai India; ^4^ Prosthetic Dental Science Department, Faculty of Dentistry Najran University Najran Saudi Arabia

**Keywords:** endodontics, guided tissue regeneration, periapical lesion, periradicular/periapical surgery, root canal therapy

## Abstract

Proper removal of the diseased tissue, debriding the canal system, and sealing the defect or cavity, the surgeon prevents or reduces the spread of microorganisms within the periradicular tissues. Treatment modalities following the failure of root canal treatment (RCT) are root canal retreatment (ReRCT). Regeneration of periapical defects may have a significant problem in periradicular surgery. In such circumstances, the gingival connective tissue can proliferate, or the oral epithelium can migrate into the defect, preventing the development of normal trabecular bone. Hard tissue can be restored using guided tissue regeneration (GTR) in conjunction with endodontic treatment for endodontic‐periodontal lesions. Treatment of large periapical defects using GTR increases overall treatment success.

## Abstract 2

Regeneration of periapical defects may have a significant problem in periradicular surgery. In such circumstances, the oral epithelium can migrate into the defect, preventing the development of normal trabecular bone. Hard tissue can be restored using guided tissue regeneration (GTR) in conjunction with endodontic treatment for endodontic‐periodontal lesions.

## 1 INTRODUCTION

Most periapical radiolucent lesions can be healed usually with conventional root canal treatment (RCT).^1^ Treatment modalities following the failure of RCT are root canal retreatment (ReRCT), apicoectomy, or extraction.^2^ In reports of apicoectomy after 1–10 years, the success rate ranges from 59.1% to 93%. In retreatment after 2–10 years, the success rate ranges from 42.1 to 86%, with higher success rates attributed to techniques and materials.^3^

By removing the diseased tissue, debriding the canal system, and sealing the defect or cavity, the surgeon prevents or reduces the spread of microorganisms within the periradicular tissues. Regeneration of periapical defects may have a significant problem in periradicular surgery. In such circumstances, the gingival connective tissue can proliferate, or the oral epithelium can migrate into the defect, preventing the development of normal trabecular bone. Hard tissue can be restored using guided tissue regeneration (GTR) in conjunction with endodontic treatment for endodontic‐periodontal lesions. Treatment of large periapical defects using GTR increases overall treatment success.^4^ Using GTR in endodontic surgery with through‐and‐through lesions that involve both the buccal and palatal alveolar cortical plates is recommended.^5^

## 2 CLINICAL PRESENTATION

**FIGURE 1:**
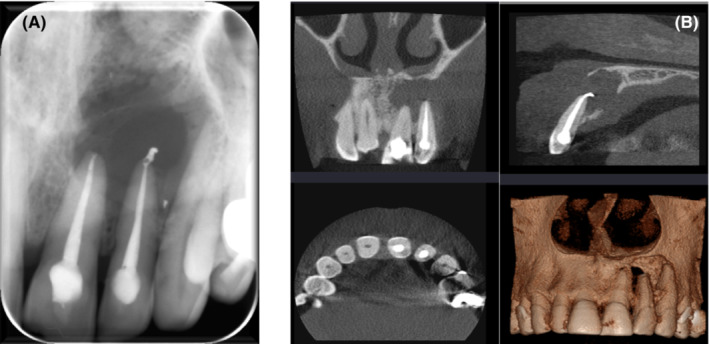
Preoperative radiograph (A) Periapical Radiograph, (B) The lesion on CBCT.

A 51‐year‐old male patient was referred by his general dental practitioner (GDP) for consultation and treatment if necessary regarding a problem related to previously root‐treated upper left central and lateral incisors (UL1 and UL2). The patient complaint that he has a concern about a swelling related to UL1 & UL2. The patient has no pain or discharge associated with these teeth. This swelling was first noticed 1 year ago and has gradually increased in size. The UL1 and UL2 have been conventionally root canal treated by endodontist specialist approximately 2 years ago followed by resin bonded composite restorations. The patient is allergic to penicillin. He is not a smoker. The extraoral examination did not reveal abnormality detected. No sinus tract or fistula was detected. Investigation of teeth UL1 and UL2 showed satisfactory coronal seal, normal probing depth more than 3 mm. Both teeth were tender on percussion and palpation. Intra‐oral periapical (IOPA) radiographic revealed radiopaque coronal restoration, radiopaque acceptable root canal filling with extruded sealer (sealer puff) on UL2, and evidence of large radiolucency around the apex (Figure 1A). CBCT also showed a large size lesion apical to UL1 and UL2 which extends from the buccal plate to the palatal bone, through and through lesion, (Figure 1B). The diagnosis of these teeth is previously treated tooth with symptomatic apical periodontitis on UL1 and UL2. After discussion with the patient, the treatment option was apical microsurgery of UL1 and UL2 using a tissue‐guided graft.

### 2.1 Procedure

Preoperative explanations of the apical surgery procedures, benefits, and risks such as vertical root fracture, bleeding, swelling, pain, and gingival recession were discussed with the patient. The consent form was signed. The patient was informed to take analgesic tablets before the surgery. The crown root ratio from the radiograph is (1:2). Probing depth at the surgical site (UL1 and UL2 and UL3) ranges between 1 and 2 mm, thick gingival biotype (probe transparency test), and medium (average) upper smile line. The patient confirmed that he had her breakfast and analgesic tablet (Paracetamol 500 mg) before surgery time. Three cartridges of 2.2 ml 2% lidocaine with 1:100000 epinephrine (Xylocaine®) were administrated to anesthetize the surgery field (as infra‐orbital nerve block and nasopalatine nerve). Two cartridges were used for labial injections and one for palatal injection.

**FIGURE 2:**
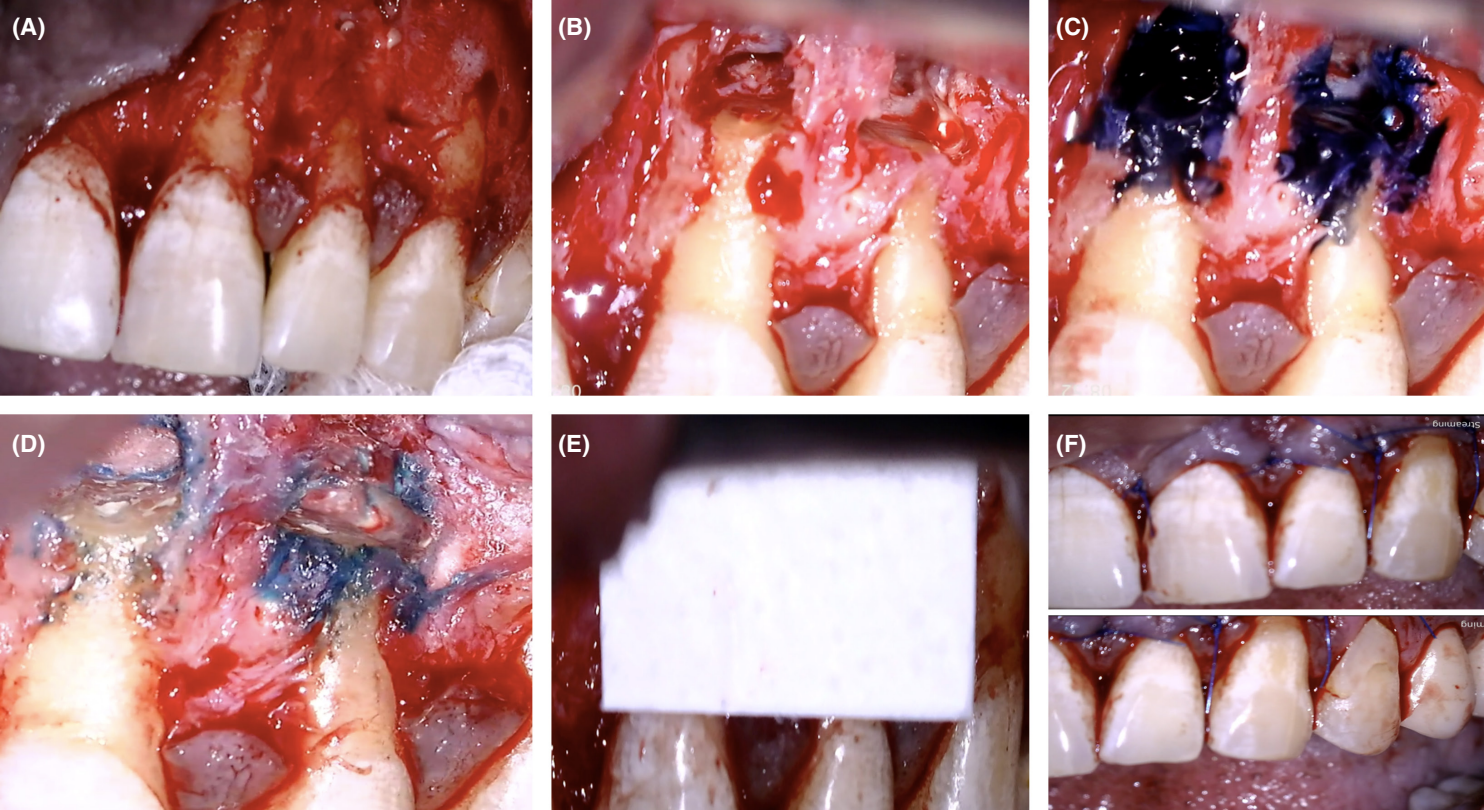
(A) Flap elevation and exposing of the bony defect on UL2, (B) Root resection to UL1 & UL2, (C) UL1 & UL2 apical root examination (stained with Methylene‐blue®), (D) MTA condensed within the retrograde preparation cavities, (E) Placement of collagen membrane, (F) Flap closure and suturing.

After 15 min from the administration of the local anesthetic, a rectangular papillary‐based design was made for two vertical releasing incisions and a horizontal incision extending from the distal surface of tooth 11 to the distal surface of tooth 23 using microblade SM64 (Swann‐Morton). The flap was carefully raised using Periosteal elevators. The flap was gently retracted with an Austin retractor; constant bone contact was kept along the surgical procedure to prevent soft tissue trauma (Figure 2A). Further apical extension of vertical releasing incisions to allow flap retraction with tensionless. The flap was kept moist during the operation.

There was no sign of vertical root fracture on UR1 and UR2; however, bone fenestration was present on the apical part with evidence of coronal reduction of bone height. Round bur with rear exhaust high‐speed surgical handpiece was used to create bone crypt on the labial side of UR1 only and the saline was used as a coolant during the osteotomy to reduce the frictional heat associated with the use of bur. Apical soft tissue was excised entirely (enucleated) using surgical curette, under the high magnification of dental operating microscope (DOM) (Global®), immediately placed in 4% formal saline, and sent to the histopathology laboratory in the school for histopathological examination/analysis. More debridement and curettage at the defect site were carried out until the root tips became clear.

To improve visualization/control hemostasis, sterile ribbon gauze was cut into six small strips and socked into local anesthetic solution (2%‐Xylocain®, 1:100000 adrenaline). The strips were packed into the osteotomy site and replaced as required throughout the surgical procedure. The apical 3 mm of root end was measured by using Williams's periodontal probe. Then measured root tip of UR1 was resected at 90° of tooth long axis using Impact‐Air 45® handpiece (SybronEndo) and round‐bur (Hu‐Friendy), and normal saline was used as coolant (Figure 2B). Methylene‐blue® (BDH‐UK) to stain the resected root tip for further inspection under high magnification Dental Operating Microscope (DOM) (Global®) in the purpose of finding out the presence of any apical root fractures /cracks. No evidence of any defects apically (Figure 2C). An apical cavity of 3 mm depth was prepared by using ultrasonic root‐end tips (KiS‐2D tip). Continuous irrigation by saline to keep osteotomy site/retrograde cavity clean.

During retrograde filling, hemostasis control was performed by applying six small strips of sterile‐ribbon gauze soaked into local‐anesthetic solution (2%‐Xylocain®, 1:80000 adrenaline).The strips were packed into the osteotomy site and replaced as required throughout the surgical procedure. Then the prepared cavities were dried using sterile gauze and then filled with mineral‐trioxide‐aggregate (ProRoot®‐MTA). MTA was mixed according to the manufacturer's instructions and placed into slots of Lee block to be carried by an instrument (micro‐plugger) and then condensed within the retrograde preparation cavities (Figure 2D). GTR technique was used in this case by using collagen membrane and placing it over bony crept (Figure 2E).

### 2.2 Flap repositioning and suturing

**FIGURE 3:**
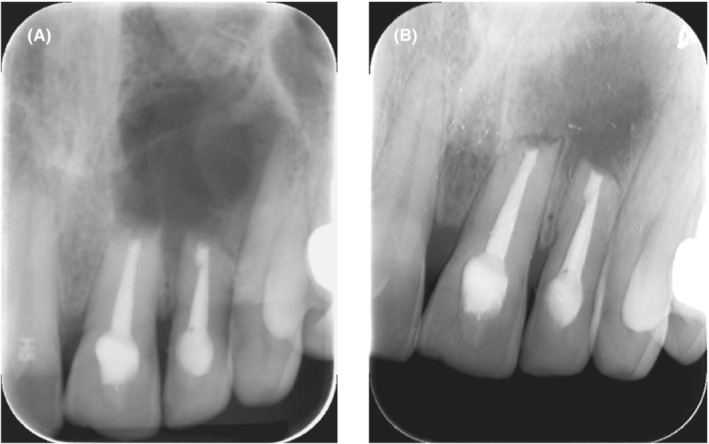
(A) Postoperative radiograph of UL1 & UL2, (B) 1 year follow up radiograph.

Irrigation and moistening of the reflected flap with normal saline and then repositioning the wound edges with gentle pressure. Then an intraoral periapical radiograph was taken to assess the quality of retrograde filling. Eight interrupted sutures using monofilament polypropylene (Prolene) 5–0 and 6–0 sutures (reverse cutting needle) were placed using a Castro Viejo needle holder and scissors (Figure 2F). Then the tissue was gently compressed for a few minutes (5 min) with saline‐moistened gauze. The postoperative periapical radiograph showed a satisfactory root‐end resection with the well‐condensed retrograde apical plug of MTA (Figure 3A).

## 3 POSTSURGICAL REVIEWS

### 3.1 One week later

The patient reported mild discomfort and swelling during the first 2 days following the surgery. There were good signs of soft tissue healing. Sutures were removed, and oral hygiene instructions were emphasized.

### 3.2 One year follow up

UL1 and UL2 were asymptomatic with no signs of infection. Periapical radiography showed almost healed at UL1 and healing process at UL2 (Figure 3B).

### 3.3 Two years follow up

**FIGURE 4:**
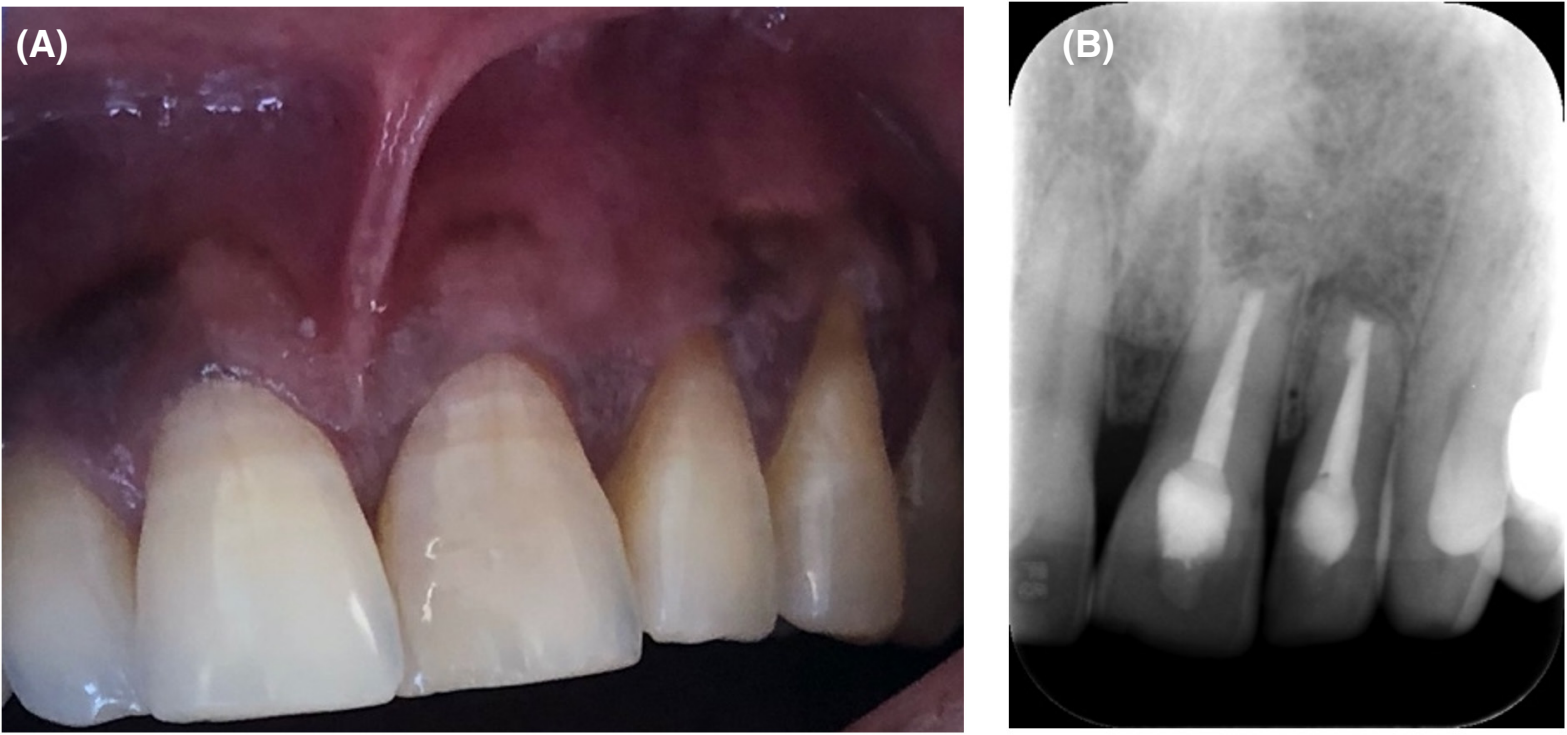
(A) Soft tissue healing, (B) 2 years follow up periapical radiograph.

UL1 and UL2 were asymptomatic with no signs of infection. The soft tissue healed (Figure 4A). Periapical radiography showed healed at UL1 and healing process at UL2 (Figure 4B).

## 4 DISCUSSION

Posttreatment endodontic disease (PTED) is defined as “the presence of an inflammatory peri‐radicular lesion in a previously root‐filled tooth when the lesion no longer can be assumed to be undergoing healing.”^6^ Intraradicular infection, extraradicular infection, true cysts, cholesterol crystals, or foreign body reactions causes PTED.^7^ Since the apical ramifications are difficult to be disinfected during the ReRCT, most PTED cases are caused by intraradicular infections that are thought to be caused by bacterial biofilms in the apical ramifications.^8^, ^9^ The UR1 and UR2 experienced periapical radiolucency, swollen, painful episodes preoperatively. Also, the root canal treatment was performed by endodontist specialist. Considering these reasons and the patient autonomy, a treatment plan for peri‐radicular surgery was carried out for the UR1 and UR2. Keeping the flap tissue moist with direct irrigation of sterile saline during the surgery procedure prevents tissue dehydration and shrinkage of the raised tissue.^10^

Using modern endodontic surgery such as the dental microscope, illumination, ultrasonic retro‐tips for root endo canal preparation showed a significant positive impact on endodontic surgery outcomes and MTA as retrofilling material.^11^ A papilla‐based incision flap was selected which chose as it has a favorable outcome in minimizing the gingival recession compared to full‐thickness mucoperiosteal flap including the interdental papilla.^12^ Using monofilament sutures has shown less plaque accumulation and bacterial colonization compared to multifilament sutures.^12^, ^13^ In addition, the use of nonresorbable monofilament sutures (size of 5‐0 and 6‐0) for endodontic surgery cases is based on the recommendation of the Royal College of Surgeons of England. The purpose of compression of repositioned flap tissue with moist gauze is to create a thin layer of fibrin between flap tissue and bone, reduce haematoma, swelling, and promote healing.^10^

The use of GTR techniques has been proposed as an adjunct to endodontic surgery in order to promote bone healing.^14^, ^15^ GTR techniques favorably affected the outcome of surgical endodontic treatments in cases of large periapical lesions (>10 mm) and through‐and‐through lesions.^4^ It has been stated in recent study^16^ that future studies should be conducted to investigate the recommendation of utilizing GTR in further with apicomarginal defects and through‐and‐through lesions. Accordingly, GTR was used based on the studies that recommend using GTR with through‐and‐through lesions. The use of the bioactive membrane in endodontic surgery should be considered to best restore the attachment apparatus to the tooth and prevent the down growth of a long junctional epithelium, which results in scar tissue healing.^17^

The periapical region lesion in this case was successfully treated and remained stable at 2 years. Furthermore, the follow‐up X‐ray illustrated good signs of healing of the periapical lesion at UL2 and healed at UL1 according to AAE. Although Azim et al^16^ found that the outcome may differ when following up with CBCT, it has not been justified to in the guidelines to follow up with CBCT. Also, the outcome in the previous study was slightly difference between using CBCT and periapical, 88%, 86% respectively. Accordingly. the periapaical radiograph is still standard of care for routine patients.

## AUTHOR CONTRIBUTIONS

BA conceived, planned the case report, carried out the clinical procedure for this case report. MIK and OA planned and carried out the simulations. BA and MIK took the lead in writing the manuscript and preparing the first draft. MIK and OA took the lead in editing the manuscript and preparing the final draft. All authors provided critical feedback and helped shape the research, analysis, and manuscript.

## CONFLICT OF INTEREST

All authors disclose that there is no actual or potential conflict of interest including any financial, personal, or other relationships with other people or organizations.

## CONSENT

Written informed consent was obtained from the patient to publish this report in accordance with the journal's patient consent policy.

## Data Availability

The (clinical pictures and radiographs) data used to support the findings of this study are included within the article.
